# Seeking a bridge between language and motor cortices: a PPI study

**DOI:** 10.3389/fnhum.2013.00249

**Published:** 2013-06-07

**Authors:** Marta Maieron, Dario Marin, Franco Fabbro, Miran Skrap

**Affiliations:** ^1^Fisica Medica, Azienda Ospedaliero Universitaria Santa Maria Della MisericordiaUdine, Italy; ^2^IRCCS “E. Medea”San Vito al Tagliamento, Italy; ^3^Dipartimento di Scienze Umane, Università degli Studi di UdineUdine, Italy; ^4^Unità Operativa di Neurochirurgia, AOUD Santa Maria della MisericordiaUdine, Italy

**Keywords:** language network, sensorimotor cortex, connectivity, PPI, tumor patiens, healthy controls

## Abstract

The relation between the sensorimotor cortex and the language network has been widely discussed but still remains controversial. Two independent theories compete to explain how this area is involved during action-related verbs processing. The embodied view assumes that action word representations activate sensorimotor representations which are accessed when an action word is processed or when an action is observed. The abstract hypothesis states that the mental representations of words are abstract and independent of the objects' sensorimotor properties they refer to. We combined neuropsychological and fMRI-PPI connectivity data, to address action-related verbs processing in neurosurgical patients with lesions involving (*N* = 5) or sparing (*N* = 5) the primary motor cortex and healthy controls (*N* = 12). A lack of significant changes in the functional coupling between the left M1 cortex and functional nodes of the linguistic network during the verb generation task was found for all the groups. In addition, we found that the ability to perform an action verb naming task was not related to a damaged M1. These data showed that there was not a task-specific functional interaction active between M1 and the inferior frontal gyrus. We will discuss how these findings indicate that action words do not automatically activate the M1 cortex; we suggest rather that its enrolment could be related to other not strictly linguistic processing.

## Introduction

There is an important debate concerning the neural processes underlying semantic representations of action words (Kemmerer and Gonzalez-Castillo, 2010). The processing of sentences and words that describe body part movements and actions has been shown to activate the sensorimotor areas of the brain, in addition to the classical language-related regions (Hauk et al., 2004; Buccino et al., 2005; Pulvermuller, 2005; Pulvermuller et al., 2005a; Tettamanti et al., 2005; Aziz-Zadeh et al., 2006; Tomasino et al., 2007; Tettamanti et al., 2008; Tomasino et al., 2008; Boulenger et al., 2009). Although it has been demonstrated that the motor system is activated during action word processing, some issues remain open for discussion, e.g., for an overview see (Willems and Hagoort, 2007); in particular there is debate on the nature of such motor activation (Mahon and Caramazza, 2005, 2008). Theories of embodied cognition argue that conceptual representations are modality-dependent and built from sensory and motor experiences (e.g., Barsalou, 1999; Gallese and Lakoff, 2005; Barsalou, 2008). Another view suggests that sensory-motor simulation is involved in linguistic processing depending on the task, on the strategies and on the context (Tomasino et al., 2007; Mahon and Caramazza, 2008; Postle et al., 2008; Tomasino et al., 2008; Papeo et al., 2009; Raposo et al., 2009; Tomasino et al., 2010; Willems et al., 2010; Papeo et al., 2012b; Tomasino et al., 2012).

Deficits in processing action-related stimuli have been reported in several studies involving patients with diseases affecting the motor system, e.g., Parkinson's disease (Boulenger et al., 2008), motor neuron disease (Bak and Hodges, 2001) and stroke involving the left hemisphere (Neininger and Pulvermuller, 2001, 2003; Kemmerer et al., 2010; Arevalo et al., 2012; Papeo et al., 2012a). Other studies, however, showed that lesions to the motor cortex do not predictably cause deficits in action word processing (De Renzi and di Pellegrino, 1995; Saygin et al., 2004; Mahon et al., 2007; Negri et al., 2007; Mahon and Caramazza, 2008; Tomasino et al., 2012). An important point in the above mentioned studies is the extent and the location of the lesions. Especially for studies involving patients with stroke, the deficit in action word processing was found to be associated with several regions across the left hemisphere and not solely with the premotor/motor or the somatosensory regions (Neininger and Pulvermuller, 2001, 2003; Kemmerer et al., 2010; Arevalo et al., 2012; Papeo et al., 2012a). Patients with relatively circumscribed lesions invading the motor areas of the brain e.g., a neurosurgical lesion (Tomasino et al., 2012) offer the possibility to specifically address the role of the sensorimotor cortex in action-related word processing. The fact that the lesions to the M1 cortex do not predictably cause deficits in action word processing is in accordance with a large body of literature addressing the neural basis of semantic memory (e.g., Vandenberghe et al., 1996; Rogers et al., 2004; Pobric et al., 2007; Binney et al., 2010; Lambon Ralph et al., 2010; Visser and Lambon Ralph, 2011). These studies used a variety of experimental approaches, such as computational models of semantic representation (Rogers et al., 2004), repetitive transcranial magnetic stimulation (rTMS) of the anterior temporal lobe, classical neuropsychological studies of patients with semantic dementia (Lambon Ralph et al., 2010), distortion-corrected fMRI, PET H_2_O (e.g., Vandenberghe et al., 1996; Binney et al., 2010; Visser and Lambon Ralph, 2011) and probabilistic tractography (e.g., Binney et al., 2012). Taken together, these studies indicate that concept representations reflect the conjoint action of modality-specific sources of information, such as the motor-related semantic associations between the words and the action in the case of action-related verbs processing, as well as a transmodal hub which is required in order to form “coherent” concepts (Lambon Ralph et al., 2010). For instance, the computational models indicate that the representational hub plays an important role in concept creation; moreover, in patients with semantic dementia the neuropsychological data show that damage to the ventrolateral anterior temporal regions generates a selective yet considerable degradation of conceptual knowledge (Lambon Ralph et al., 2010), which is not affected by damage to *modality-specific association* regions.

Although previous studies also point to an involvement of the motor system in processing action verbs (e.g., Tettamanti et al., 2005; Aziz-Zadeh et al., 2006), in the present study we were primarily interested in the role of the (left) M1 cortex, given that resection of lesions in the sensorimotor cortex is rare. We used a block design fMRI experiment where 12 healthy participants and 10 neurosurgical patients with lesions involving or sparing the primary motor cortex performed an action-verb generation task. It has been suggested that the response to an object picture is a valid way to address the relationship between the neural substrates of language processing and the motor system (Peran et al., 2010). In that study, authors found activation in the pre- and post-central gyrus during action-verb generation (Peran et al., 2010). Similarly, other authors found activation for the semantic generation task in proximity of the hand or foot motor cortex (Esopenko et al., 2012). It has been argued that action-related representations are involved in tasks implying active semantic search during the generation of action verbs (Peran et al., 2010). For these reasons, we used a verb generation task in response to pictures; this task was designed to suit even cognitively impaired subjects, since it is known that subjects are faster at performing semantic tasks with pictures than words (Chainay and Humphreys, 2002) and that pictorial stimuli have privileged access to manipulation knowledge compared to word stimuli (Thompson-Schill et al., 2006). In addition, it is held that to generate a verb in response to a picture one must select concepts that are associated with the object picture. In our experiment, we addressed two main points: firstly, the anatomo-functional correlates of action-verb generation task in healthy participants and in neurosurgical patients with lesions involving or sparing the M1 cortex and the main differences between their activations under classical General Linear Model assumptions. Secondly, to highlight the results, we also assessed the functional connectivity, using psycho-physiological interactions (PPI) (Friston et al., 1997).

The embodied view suggests that the linguistic processing of action-related words and the M1 cortex interact (Hauk et al., 2004) which implies an increase of the functional connectivity between language-related areas and motor-related areas. For instance, the comprehension of action-related sentences should be associated with a relatively stronger functional integration between the perisylvian regions and M1. There is a limited number of studies addressing how do language-related areas and motor-related areas functionally talk to each other. In one of those studies, authors used dynamic causal modeling (DCM) to analyze fMRI data during a listening task involving action- and non-action related stimuli presented first as affirmative and then negative sentences (Tettamanti et al., 2008). It was found that within the action representation system, the modulatory effects of action-related vs. abstract sentences were stronger for affirmative than negative sentences. Another result of the study was that the degree of functional integration between the left inferior frontal gyrus and the left fronto-parieto-temporal system, including the dorsal premotor cortex, the supramarginal gyrus, and the left posterior inferior temporal gyrus, was more positive for processing action-related vs. abstract sentences (Tettamanti et al., 2008). Authors argued that their results complement the findings of more classical analyses of functional specialization underlying action-related conceptual representations (Pulvermuller, 2005) and that they are in agreement with previous studies showing a more positive functional integration among the left fronto-parieto-temporal region for action-related semantic processing, in particular for pictures of tools vs. animals (Vitali et al., 2005; Noppeney et al., 2006). In a further study, DCM was used to test the semantic domain-specific patterns of the functional integration between the language and the modal semantic brain regions during the listening of either action-related or abstract sentences (Ghio and Tettamanti, 2010). Authors found that the left superior temporal gyrus was more strongly connected with the left-hemispheric action representation system, including sensorimotor areas when participants processed for action-related sentences, and with the left infero-ventral frontal, temporal, and retrosplenial cingulate areas for abstract sentences. Furthermore, authors found that causal modulatory effects were exerted by the perisylvian language regions on peripheral modal areas, and not vice versa (Ghio and Tettamanti, 2010). Lastly, other authors used psychophysiological interaction analysis (PPI) for testing whether the functional integration between the auditory brain regions and the perception/action areas is modulated by a context in which words with both motor and visual properties are presented (van Dam et al., 2012a). Results showed that the bilateral superior temporal gyrus was more strongly connected with brain regions relevant for coding action information when subjects were processing action color words (as compared to abstract words), and for action color words presented in a context that emphasized action vs. a context that emphasized color properties. Authors argued that their results corroborate the view that language representations are flexible and context-dependent (van Dam et al., 2012a).

In the present study, we first measured the functional connectivity between language-related areas and M1 as calculated by psycho-physiological interactions (PPI) in healthy controls and in patients whose lesion affected the motor areas. Neurosurgical patients were studied before surgery. PPI analyses were performed on the left M1 (as revealed by the motor localizer task) and on the left inferior frontal gyrus (Pars Opercularis, as revealed by the whole brain analysis of the main fMRI experiment) as seeds to assess the areas with increased connectivity with the left primary motor area and with the left inferior frontal gyrus during action-related word processing. PPI analysis is used to explain the neural responses in one brain area in terms of the interaction between influences of another brain region and a cognitive process (here: action-related word processing). According to the embodied view, we should expect that the functional connectivity between the language-related areas and M1 is reduced in patients who show a significantly decreased ability in processing action-related words and whose lesions affect the motor areas. On the contrary, a lack of functional connectivity changes would support the view that sensory-motor activity is not necessary but rather accessory to linguistic processing (Tomasino et al., 2007; Mahon and Caramazza, 2008; Postle et al., 2008; Tomasino et al., 2008; Papeo et al., 2009; Raposo et al., 2009; Tomasino et al., 2010; Willems et al., 2010; Papeo et al., 2012b; Tomasino et al., 2012). In addition, showing that lesions to the M1 cortex do not degrade action-related word processing complements a large body of literature addressing the neural basis of semantic memory, and showing that although concept representations reflect the conjoint action of modality-specific sources of information (Lambon Ralph et al., 2010), degradation of conceptual knowledge is generated following damage to the ventrolateral anterior temporal regions (and not to the modality-specific association regions) (Lambon Ralph et al., 2010).

## Materials and methods

### Participants

#### Patients

**Figure 1:**
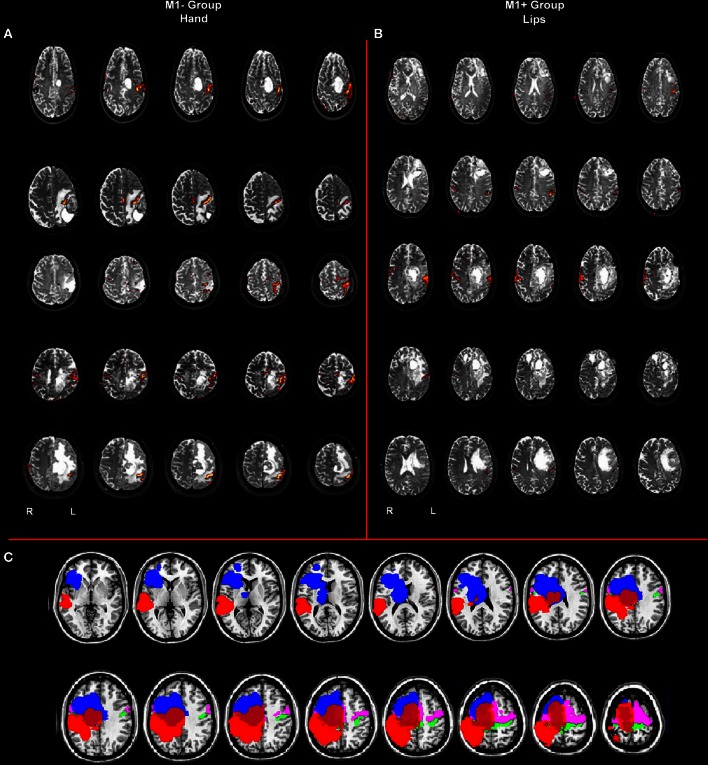
**(A)** Whole brain analysis results for the group of patients with lesions involving M1 (M1−) performing hand clenching movements vs. rest and **(B)** activation maps for the group of patients with lesions sparing M1 (M1+) performing lip movements vs. rest. The two types of movement have been selected in this image to highlight the close location of the M1− to the hand representation area, as evidenced by the activation cluster, and the vicinity of the M1+ to the lip representation area, as evidenced by the activation cluster. Data were thresholded at *p* < 0.05 cluster corrected (*Z* > 2.3). **(C)** Overlapping of the ROIs drawn on the patients' lesions after normalization (in blue for the M1+ and in red for the M1−) and of the mask created by using the Anatomy Toolbox and the maximum probability maps (MPS) of the left and right M1 (in green) and of the left and right Pm cortex (in pink).

Ten right-handed neurosurgical patients (5 M, 5 F) whose tumor involved the left hemisphere either sparing (*N* = 5, 3 F, mean age 48.2 years, range 31–62) or involving (*N* = 5, 2 F, mean age 43.6 years, range 26–58) the primary motor cortex (M1+ and M1−, respectively), gave informed consent to participate in the study. All participants were native Italian speakers, had normal or corrected-to-normal vision and reported no history of psychiatric disease nor drug abuse. All the patients participated in the study before surgery (see Figure 1).

#### Control group (healthy volunteers)

The control group consisted of twelve right-handed volunteers (6 F, 6 M, mean age 48 years, range 35–60) were selected from a pool of data on healthy controls previously published (Tomasino et al., 2013) and were matched in education level with our patient sample (range 8–17 years of education) (Healthy control Group; HC). All the participants were native Italian speakers, with no history of neurological nor psychiatric disorders and with no structural brain abnormalities.

### Neuropsychological examination

Each patient was submitted to a neuropsychological battery one day before fMRI. Handedness was evaluated with the Edinburgh Handedness Inventory (Oldfield, 1971). The neuropsychological evaluation included tests assessing non-verbal intelligence (Basso et al., 1987); verbal short-term digit span memory (Orsini et al., 1987); oral apraxia (Spinnler and Tognoni, 1987); ideomotor apraxia (De Renzi et al., 1980) and language. The following language tasks were performed: Token test (De Renzi and Faglioni, 1978); verbal fluency (Novelli et al., 1896); noun and verb naming (Miceli et al., 1994). Using the SPSS software for Windows, version 15 (SPSS Inc, Chicago, Ill, US), a non-parametric *t*-test (Mann–Whitney U test) was performed to evaluate the accuracy differences between groups of subjects.

### Functional MRI and DTI acquisitions

MRI data were collected on a whole-body 3 Tesla Philips Achieva (Best, Netherlands) MRI scanner equipped with a SENSE-Head-8 channel coil. Functional runs were acquired using a T2^*^ BOLD—sensitive gradient-recalled EPI sequence; imaging parameters were as follows: TR = 2500 ms; TE = 35 ms; 90° flip angle; SENSE reduction factor in phase encoding direction = 2; FOV = 23 × 23 cm; 128 × 128 image matrix, yielding an in-plane voxel size of 1.8 × 1.8 mm; 34 axial slices, slice thickness = 3 mm; no gap. Head motion was reduced by a foam custom built head cushion around the subject's head. The MR scanner was allowed to reach a steady state by discarding the first four volumes in each scan series, since they were collected before equilibrium magnetization was reached. Anatomical T1-Weighted images MPRAGE were also acquired (190 sagittal slices; TR = 8.1007; TE = 3.707 ms; flip angle 8°; FOV = 24 cm; voxel size 1 × 1 × 1 mm) to obtain structural three-dimensional (3-D) volumes. In addition we acquired DTI data using a single-shot EPI sequence (TR/TE = 8800/74 ms, bandwidth = 1287 Hz/pixel, flip angle = 90°, FOV = 224 × 224 cm, in-plane resolution 1.8 × 1.8 mm). The gradient directions were uniformly distributed on a sphere. Diffusion gradients were applied along 64 non-coplanar axes, using a *b*-value of 0 and 1000 s/mm^2^. Seventy contiguous axial slices were acquired, with a thickness of 1.5 mm, with no gap. Total time for diffusion tensor MR imaging was 13 min 56 s.

### Task and paradigm

Participants performed three runs of one task each: two motor tasks and a language task organized in a boxcar paradigm, composed of baseline and activation periods (*15 s on −15 s off*) the active conditions were repeated four times. In the motor run, participants were required to perform repetitive movements of the lips, and, in the second motor run, clenching hand movements. Instructions about the beginning, the end, and the side of the movement were visually cued during the fMRI acquisition for a total duration of 135 s for the lip localizer and 255 s for the hand localizer. In the language run participants were instructed to silently generate verbs evoked by visually presented objects; in each active block (*N* = 4) seven items were presented for a total duration of 135 s (see Appendix). The pictures were selected from the Snodgrass and Vanderwart's set of pictures (Snodgrass and Vanderwart, 1980) (mean word length 6.8 ± 2.2; length in syllable 2.8 ± 0.89; frequency 1.4 ± 1.6). For both experiments, participants were instructed to relax and remain still, to keep their arms aligned with the sides of the body, and to breath normally. Stimuli and instructions were presented through a VisuaStim Goggles system (NordicNeuroLab, Bergen, Norway) equipped with the Presentation® software (Version 9.9, Neurobehavioral Systems Inc., CA, USA)

### DTI data analysis

Images were analyzed using DTIStudio, version 3.0.3 (2010), (Kennedy Kriger Institute, Baltimore, MD, USA) software obtaining main eigenvector, fractional anisotropy (FA) maps and color maps generated with conventional coding-color (Pajevic and Pierpaoli, 1999). Deterministic tractography was performed in all patients and subjects to reconstruct superior longitudinal fasciculus (SLF) using the fiber assignment by continuous tracking method (Mori et al., 1999, 2005) in both hemispheres. An FA threshold of 1.5 and a turning angle >45° were used as criteria to start and stop tracking. The SLF tract was reconstructed using a multi-ROI approach (Wakana et al., 2007): the first ROI was placed on a coronal view at the level of the middle of the posterior limb of the internal capsule on the intense triangle-shape green structure which identified the SLF tract. The second ROI was even placed on a coronal slice at the splenium of corpus callosum to select the descending branch of the tract. For all the tracts reconstructed, eventual contaminating fibers were removed.

Tracts were then classified as unchanged, displaced or infiltrated/disrupted as described in previous articles (Witwer et al., 2002; Jellison et al., 2004). Unchanged reconstructed tracts exhibited normal anisotropy, location and orientation, compared with homologous contralateral tracts. Displaced tracts had a normal or any slightly reduced anisotropy and showed abnormal location or trajectories when compared with contralateral hemisphere. Infiltrated tracts showed considerably decreased FA with altered color patterns on directional maps. Disruption represented an extreme case of infiltration, with near-zero anisotropy due to destruction of fibers and interruption of DTI tractography reconstruction. Fiber tract FA and number of reconstructed fibers were evaluated between groups using a non-parametric *t*-test (Mann–Whitney U test) by SPSS software.

### fMRI data analysis

Image analysis was performed on each subject's data using FSL (FMRIB'S Software Lybrary, www.fmrib.ox.ac.uk/fsl). Data were skull stripped with BET (Smith, 2002), motion corrected with MCFLIRT (Jenkinson et al., 2002), smoothed with gaussian kernel (5 mm FWHM), and registered with FLIRT (Jenkinson et al., 2002) to standard MNI152 template image supplied by the Montreal Neurological Institute using the affine transformation method. We paid particular attention to patient's normalization in order to ensure a correct alignment with the template (Brett et al., 2001): first, for all patients a lesion mask was drawn and linearly registered with FLIRT on the T1-W image using affine transformation parameters with a normalized mutual information cost function. Second, each lesion mask previously registered on the T1-W image was non-linearly registered on the template with FMRIB's non-linear image registration tool (Andersson et al., 2010) using the transformation parameters derived by registering the T1-W image on the template. The nearest neighbor interpolation method was used in both stages. Two observers (M.M. and D.M.) independently checked all the co-registered lesion masks and an agreement was found in all cases. The task timing was convolved with the standard gamma variate function implemented in FSL (lag, 6 s; width, 3 s), and the fMRI signal was then linearly modeled (Worsley and Friston, 1995) on a voxel-by-voxel basis using a general linear model (GLM) approach, with local autocorrelation correction (Woolrich et al., 2001) to calculate the subject-specific parameter estimates for each event type. The estimated translation and rotation parameters were added as confounds in the model. At the single subject level, specific effects were tested by applying linear contrast to the parameter estimates for each event (active vs. rest) and the calculated *Z* statistic images were thresholded at the whole-brain level using clusters determined with *Z* > 2.3 voxelwise thresholding and a family-wise error-corrected cluster significance threshold of *p* = 0.05 (Worsley, 2001). Only for the language task, we performed a higher-level random effects group analysis, assessing the consistency and differences of the language network between healthy controls (HC), patients with a lesion involving or sparing the motor cortex.

In addition, to estimate the functional connectivity during the action naming task, two psycho-physiological interaction (PPI) analyses (Friston, 1997) were conducted in order to test for significant PPIs with activity in Broca's area and in the primary motor area, assessing whether those two areas interact during the language task execution. PPI analysis simply tells us which voxels across the whole brain increase their signal changes related to the seed ROI during and modulated by task execution. PPI analysis is a simple brain connectivity method that characterizes the activity in one brain region by interaction between another region's activity and a psychological factor, and an interregional correlation analysis (O'Reilly et al., 2012). PPI functional connectivity analysis has the capacity to detect regions whose BOLD hemodynamic response significantly covaries with the activity of selected areas during the performance of the task. Brain areas which exhibit significant covariance with the activity of selected ROIs over the time course can be considered as functionally connected to each other's by the task. Our ROIs were functionally and structurally constrained. We identify the seed ROIs from the previous subject-level GLM analysis of the language task and the hand clenching task. From the GLM results of the language task we identify the functionally activated cluster closest to the Broca's area [Areas 44 and 45 of Brodmann's cytoarchitectonic map (Dronkers et al., 2007)] for each subject and patient. We used the coordinates of the local maximum of this cluster as the centre of the seed region, defined as a sphere with a 6 mm radius. In the same way, the second ROI seed was centered on the local maximum of the motor hand area for each participant, as identified by functional analysis of the motor task. The primary motor hand area was identified as the cluster located in the precentralgyrus, structurally defined using the FSL Harvard–Oxford cortical atlas.

We performed two-step analysis: in the first level analysis, for each participant a PPI regressor was extracted. The PPI regressor was the result of the convolution of two functions: the hemodynamic-response-function-convolved task regressor (for the naming actions–baseline contrast), and the BOLD time-course of the spherical seed ROI. This regressor was used to identify the individual effect of task modulation on functional connectivity due to the language task. While the first level analysis involved the subjects at an individual level, the second level analysis was performed at a group level. In both analyses, *Z* statistic images where thresholded at the whole brain level using cluster determined with *Z* > 2.3 voxelwise thresholding and a family-wise error-corrected cluster significance threshold of *p* = 0.05.

PPI analysis estimates the connectivity, allowing to test whether the inter-regional correlation in neuronal activities changes significantly as a function of the task condition, *independently of activity due to task* differences. This “functional connectivity” analysis differs from the conventional activation mapping approach in that PPI reveals differential interactions between brain regions on residual variance after removing task-related effect, and hence disambiguates inter-regional connectivity from differential task effects (Friston, 1997). With PPI analysis, we tested the connectivity of Broca's area and the primary motor cortices, in order to assess how those areas interact and are functionally connected in a verb generation task.

## Results

### Neuropsychological evaluation

As reported in Table 1, the group with lesions involving M1 (M1−) significantly differed from the group with lesions sparing M1 (M1+) at verb naming (Mann–Whitney U test, *Z* = −2.66, *p* = 0.008). While M1− had a performance within the normal range (mean 27.4/28), M1+ had a performance significantly below the normal range (mean 22.6/28). Note that the cut-offs of noun naming and verb naming are 28 and 26, respectively (Miceli et al., 1994), and all the single M1+ patients scored below the normal range at the verb naming task. Possible noun-verb naming dissociations were not the subject of the present study, which focuses on verb naming, irrespective of noun naming performance. On the remaining neuropsychological tasks we didn't find any significant difference between the groups (noun naming, *Z* = 2.132, *p* = 0.056; RCPM, *Z* = −1.786, *p* = 0.095; oral apraxia, *Z* = −0.149, *p* = 1.00; ideomotor apraxia, *Z* = −1.838, *p* = 0.095; phonological fluency, *Z* = 0.21, *p* = 0.841; Token test, *Z* = −0.346, *p* = 0.548; short-term memory *Z* = −0.346, *p* = 0.729). As to their performance at noun naming, both groups scored within the normal range (M1− mean naming nouns 29.4/30 and M1+ mean naming nouns 27.2/30, respectively, cut-off 28). Therefore, there was also a dissociation between group and type of stimulus (nouns, verbs). As to the single patient performance, each individual of the M1+ group scored: P1: 23/28, and 29/30 P2:25/28, and 29/30, P3:22/28, and 25/30, P4:23/28, and 28/30, and P5:20/28 and 25/30 at the verb and at the noun naming task, respectively. By contrast, each individual of the M1− group scored: P1: 28/28, and 30/30, P2:27/28, and 29/30, P3:27/28, and 29/30, P4:27/28, and 29/30, and P5:28/28 and 30/30 at the verb and at the noun naming task, respectively.

**Table 1:** **Clinical/demographic data and neuropsychological evaluation for the patients included in the study**.

	**M1−**					**M1+**				
	**P1**	**P2**	**P3**	**P4**	**P5**	**P6**	**P7**	**P8**	**P9**	**P10**
Lesion	F, motor, LH	F, motor, LH	F, motor, premotor LH	F, motor, premotor LH	F, motor, LH	F, Pm, frontal opercularis LH	F, Pm, frontal opercularis LH	F, Pm, LH	F, frontal opercularis, LH	F, frontal opercularis, LH
Histology	Oligoastrocytoma	Glioblastoma	Astrocytoma	Glioblastoma	Atypical Meningioma	Glioblastoma	Oligoastrocytoma	Astrocytoma	Astrocytoma	Metastatic melanoma
Tumor grade	Low grade	High grade	Low grade	High grade	High grade	High grade	High grade	High grade	High grade	High grade
Lesion volume	29,5 cc	60,8 cc	28,6 cc	28,3 cc	42,2 cc	10,3 cc	18,3 cc	45,2 cc	84 cc	28 cc
Pre-surgery	Seizures (general)	Seizures R (arm and face); paresthesia R side of the body	Paresthesia R side of the body and Seizures (general)	Seizures R (arm and leg)	Seizures R (arm); seizures (general)	Speech disorder	Seizures (mouth)	Motor weakness R side of the body	Paresthesia R (arm and leg)	Motor weakness R leg; dysarthria
Age	26	58	46	52	36	66	31	36	46	62
Sex	M	M	F	F	M	F	F	F	M	M
Handedness	R	R	R	R	R	R	R	R	R	R
Comprehension	36/36	**25/36**	**26/36**	36/36	34/36	34/36	33/36	**24/36**	36/36	**25/36**
Naming nouns	30/30	29/30	29/30	29/30	30/30	29/30	29/30	**25/30**	28/30	**25/30**
Naming actions	28/28	27/28	27/28	27/28	28/28	**23/28**	**25/28**	**22/28**	**23/28**	**20/28**
ST memory	7	5	5	6	6	6	5	6	5	6
Oral praxis	20/20	19/20	20/20	20/20	20/20	20/20	20/20	17/20	20/20	20/20
IMA	72/72	**46/72**	72/72	72/72	72/72	69/72	65/72	**42/72**	65/72	55/72
RCPM	35/36	33/36	33/36	25/36	36/36	30/36	26/36	24/36	30/36	32/36
Ph. Fluency	50	**10**	26	34	**13**	34	17	18	**15**	21

Handedness (Oldfield, 1971); language, comprehension [token test, (De Renzi and Faglioni, 1978) (cut-off score: 29)]; naming nouns and verbs [BADA, (Miceli et al., 1994) (cut-off score 28/30; 26/28)]; STM, short term memory, digit span: test from Orsini et al. (1987) (cut-off score 4); oral praxis (Spinnler and Tognoni, 1987) (cut-off score 16); IMA: ideomotor apraxia test (De Renzi et al., 1980) (cut-off score 53); raven coloured progressive matrices (Basso et al., 1987) (cut-off score 18); phonological fluency (Novelli et al., 1896) (cut-off score 17). In bold type the patient's performance below the normal range.

### DTI data

In the group of patients sparing M1 (M1+), the analysis of DTI data showed that the left Superior Longitudinal Fasciculus (SLF) was unchanged in P2 (20%), infiltrated in P1 (20%) and displaced in P3, P4, and P5 (60%). In the group with lesions involving M1 (M1−), the analysis of DTI data revealed that the left SLF was unchanged in P1, P3, and P5 (60%), and non-reconstructable in P2 and P4 (40%). The SLF tract in the right hemisphere was reconstructed for all patients. Finally, in all healthy subjects (100%) unchanged SLF tracts were reconstructed on both hemispheres (see Table 2).

**Table 2:** **Results of the DTI analysis**.

	**Lesion_side**			**Healty side**			**Classification**
	**FA**	**s**	**Numbers of fibers**	**FA**	**s**	**Numbers of fibers**	
**M1−**							
1	0.36	0.13	475	0.38	0.11	481	Unchanged
2	–	–	–	0.44	0.11	369	Distrupted
3	0.46	0.12	585	0.46	0.10	520	Unchanged
4	–	–	–	0.45	0.12	369	Distrupted
5	0.41	0.14	423	0.45	0.11	438	Unchanged
Mean	0.41	0.13	494	0.44	0.11	435	
**M1+**							
1	0.36	0.13	285	0.45	0.11	351	Infiltrated
2	0.44	0.11	218	0.49	0.13	199	Unchanged
3	0.42	0.13	584	0.43	0.11	656	Displaced
4	0.37	0.10	236	0.43	0.12	449	Displaced
5	0.48	0.15	399	0.47	0.13	483	Displaced
Mean	0.41	0.12	344	0.45	0.12	428	
**HC**							
1	0.50	0.12	846	0.47	0.10	769	–
2	0.48	0.13	626	0.46	0.12	570	–
3	0.50	0.10	646	0.46	0.12	545	–
4	0.52	0.12	584	0.47	0.11	496	–
5	0.48	0.11	534	0.48	0.13	491	–
6	0.47	0.12	673	0.50	0.12	654	–
7	0.50	0.11	565	0.50	0.10	501	–
8	0.47	0.13	632	0.46	0.10	587	–
9	0.50	0.10	763	0.49	0.11	671	–
10	0.51	0.10	578	0.49	0.11	499	–
11	0.49	0.11	584	0.47	0.10	500	–
12	0.47	0.12	873	0.47	0.10	754	–
Mean	0.49	0.11	659	0.48	0.11	586	–

The value of FA and the number of fibers of the SLF tract are shown for all the three groups. For healthy controls no classification is reported because they show always unchanged tracts between sides.

Focusing on the number of fibers, interhemispheric differences were found in healthy subjects (using a paired t test) (left side = 659 ± 111, right side 586 ± 102, *t* = 8.94; *p* < 0.001), no differences were found for M1− patients (left side = 494 ± 82, right side 435 ± 67, *t* = 0.58; *p* = 0.621) and for M1+ patients (left side = 344 ± 151, right side 428 ± 168, *t* = −2.235; *p* = 0.89). Interhemispheric asymmetry was found on the FA value for HC group(left side = 0.49 ± 0.11, right side 0.48 ± 11, *t* = 2.327; *p* = 0.040) but not for M1− (left side = 0.41 ± 0.13, right side 0.44 ± 0.11, *t* = −1.732; *p* = 0.225) and M1+ (left side = 0.41 ± 0.12, right side 0.45 ± 0.12, *t* = −2.236; *p* = 0.89).

However, the Mann–Whitney U test shows a significantly decreased value of FA and the number of fibers in the left affected hemisphere for the M1+ group (0.41 ± 0.12 and 344 ± 151) compared with healthy controls (0.49 ± 0.11 and 659 ± 111), *Z* = −2.747; *p* = 0.006 and *Z* = −2.771; *p* = 0.006.

For the M1− group (0.41 ± 0.13 and 494 ± 82) compared with healthy controls, we found a significant difference only in FA values, *Z* = 2.634; *p* = 0.008 but not in the number of fibers, *Z* = −1.878; *p* = 0.06. Moreover, FA values and number of fibers showed no differences, when comparing the two patient groups (FA: *Z* = −0.30, *p* = 0.786; numbers of fibers: *Z* = −1.64, *p* = 0.143).

### Functional MRI data

#### Group analysis of the motor tasks

The lip representation area and the hand motor area have been identified for all the patients (Figure 1), thus ruling out the possibility that a lesion affecting the motor areas could have compromised the signal change in the primary motor cortex, if any, during the linguistic task. BOLD time course was extracted from the primary motor cortex of each subject and used for PPI analysis. The motor network activated the standard hand motor region involved in the execution of movements for each subject (Table 3). Even for the group with lesions involving M1 (M1−), fMRI data analysis allows us to identify the correct position of the primary motor area, verifying that no significant displacements or absence of activation occurred.

**Table 3:** **MNI coordinates and *Z*-value group statistics for most strongly activated voxel during hand motor localizer scan**.

		**Coordinates (mm)**			**Primary motor**
	***Z*-value**	***x***	***y***	***z***	**Cortex**
HC 1	9.01	−40	−20	49	BA 4p
HC 2	10.23	−38	−24	54	BA 4a
HC 3	9.56	−32	−22	50	BA 4p
HC 4	13.25	−40	−28	52	BA 4p
HC 5	12.09	−34	−26	52	BA 4p
HC 6	12.26	−38	−22	50	BA 4p
HC 7	12.54	−45	−23	17	BA 4a
HC 8	10.86	−41	−20	59	BA 4a
HC 9	12.76	−39	−18	56	BA 4a
HC 10	11.34	−38	−16	54	BA 4p
HC 11	12.98	−37	−18	52	BA 4p
HC_12	13.02	−40	−21	53	BA 4p
M1− 1	9.76	−40	−28	50	BA 4p
M1− 2	8.73	−32	−18	52	BA 4p
M1− 3	7.98	−36	−22	52	BA 4p
M1− 4	10.73	−40	−32	50	BA 4p
M1− 5	7.98	−40	−28	46	BA 4p
M1+ 1	5.73	−40	−29	50	BA 4p
M1+ 2	7.98	−40	−31	50	BA 4p
M1+ 3	8.83	−36	−27	50	BA 4p
M1+ 4	5.42	−38	−22	48	BA 4p
M1+ 5	7.91	−32	−34	46	BA 4p

#### Group analysis of the verb generation task

**Figure 2:**
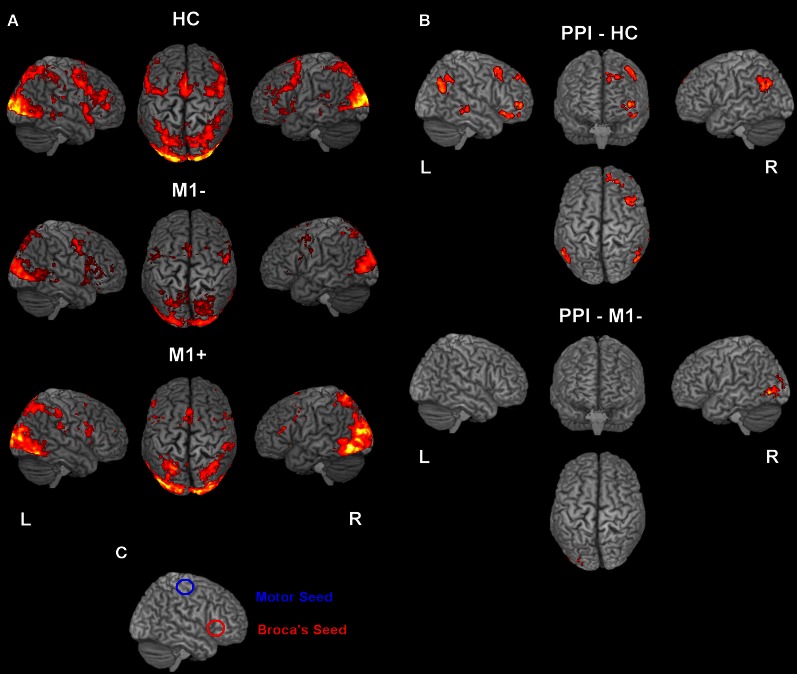
**(A)** Activation elicited by the verb generation task vs. rest (*p* < 0.05 cluster corrected (*Z* > 2.3) for healthy controls (upper row), for patients with lesions involving M1 (M1−, middle row) and for patients with lesions sparing M1 (M1+, lower row). **(B)** The image shows the activation maps generated by the PPI analysis. Brain regions showing significant increases of connectivity to the left Broca's area during verb generation task for healthy controls and for M1− are shown. For M1+ PPI analysis didn't find any area with a significant activation. **(C)** Overlapping of the seed regions (Broca's and M1 area) on a rendered 3D template.

The verb generation task (verb generation > rest) triggered a cluster of increased activity in a set of brain regions typically found in language-related tasks, including: bilaterally in the occipital lobe, bilaterally in the hippocampus, in the temporal inferior cortex, the left precentralgyrus, the SMA, and the left insula (Table 4, Figure 2A)

**Table 4:** **MNI coordinates and second level group statistics for voxels that were most strongly activated by the verb generation task vs. rest**.

			**Coordinates(mm)**				
**Cluster**	**Voxels**	***P***	***Z***	***x***	***y***	***z***	
**HC**							
1	41,145	0	17.5	−48	−84	−4	Lateral occipital pole L
			16.8	16	−102	8	Occipital pole R
			15.4	−22	−100	2	Occipital pole L
2	218	9.42E-05	5.63	64	−44	18	Supramarginal gyrus R
			3.88	66	−34	10	Superior temporal gyrus R
			3.7	66	−48	24	Angular gyrus R
			3.09	58	−38	8	Middle temporal gyrus R
3	130	0.00825	4.84	−28	50	−18	Frontal pole L
			4.84	−28	50	−18	Frontal pole L
4	129	0.00872	4.61	32	−18	2	Right putamen
			3.63	14	6	8	Right caudate
5	107	0.0306	4.53	−12	−16	4	Left thalamus
			3.31	2	−10	10	Right thalamus
**M1−**							
1	14,469	0	13.4	28	−88	20	Lateral occipital cortex R
			12.2	−40	−88	8	Occipital pole R
			11.7	−22	−92	10	Precentral gyrus L
2	3258	3.17E-39	9.31	−58	0	38	Precentral gyrus L
			6.23	−50	14	16	Inferior frontal gyrus L
3	1751	2.82E-25	6.13	−38	24	4	Insular cortex L
			5.75	−48	0	30	Precentral gyrus L
			5.14	−24	0	70	Superior frontal gyrus L
			5.11	−46	22	22	Inferior frontal gyrus L
4	1603	1.01E-23	7.93	24	−66	56	Parietal superior cortex R
			7.91	28	−64	60	Lateral occipital cortex L
5	947	3.94E-16	6.77	−2	14	56	Supplementary motor cortex L
			6.26	0	12	60	Superior frontal gyrus L
			4.84	4	18	66	Supplementary motor cortex R
6	146	0.00288	4.68	−34	46	24	Middle frontal gyrus L
			4.55	−46	48	8	Frontal pole L
7	112	0.0198	4.72	22	−32	−4	Thalamus R
			3.5	16	−40	0	Cingulate gyrus R
			3.49	32	−34	−2	Hippocampus R
			3.26	16	−30	−10	Parahippocampal gyrus R
8	109	0.0237	3.99	−26	−26	−5	Hippocampus L
			3.81	−20	−30	−10	Parahippocampal gyrus L
			3.1	−36	−34	−16	Temporal fusiform cortex L
**M1+**							
1	24,068	0	16.1	44	−72	−10	Lateral occipital cortex/occipital fusiform gyrus R
			14.2	−30	−90	6	Lateral occipital cortex/occipital fusiform gyrus L
			13.7	30	−78	−14	Occipital fusiform gyrus R
			13.5	32	−92	16	Occipital pole R
2	2887	2.07E-35	8.03	8	10	72	Superior frontal gyrus /SMA R
			7.68	0	20	56	Superior frontal gyrus R
			6.98	54	34	12	Inferior frontal gyrus, parstriangularis R
			6.67	44	20	22	Inferior frontal gyrus R
3	227	6.44E-05	5.24	−38	8	58	Middle frontal gyrus L
			4.7	−40	2	42	Precentral gyrus L
4	174	0.000836	4.6	−38	24	−6	Frontal orbital cortex/insular cortex L
			4.47	−32	38	−8	Frontal pole L
5	105	0.036	5.01	−40	52	18	Frontal pole L

HC, healthy controls; M1−, lesions involving the primary motor cortex; M1+, lesions sparing the primary motor cortex.

The contrast (verb generation > rest) in healthy controls (HC) > group with lesions involving M1 (M1−), showed significantly higher activity in the superior frontal gyrus, the precentralgyrus, the postcentralgyrus, the middle temporal gyrus, the precuneus and the lateral occipital cortex. The opposite contrast (M1− > HC) showed an increased activity in the lateral occipital cortex, the occipital pole, the inferior frontal gyrus.

**Figure 3:**
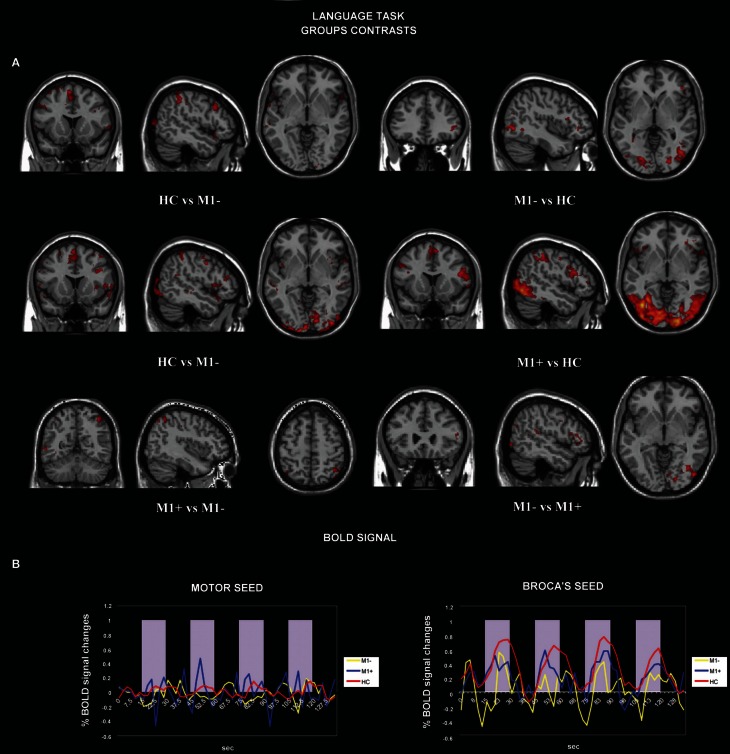
**(A)** Activation maps for whole-brain GLM analysis related to verb generation task vs. rest. The contrast data between groups are presented at a threshold of *p* < 0.05, cluster corrected for *Z* > 2.3. **(B)** Average BOLD signal time-course extracted from the motor seed ROI (left) and from the Broca's seed ROI (right) during the verb generation task are displayed for all the groups separately (M1−, lesions involving the primary motor cortex; M1+, lesions sparing M1; HC, healthy controls).

The contrast (verb generation > rest) in HC > group with lesions sparing M1 (M1+) showed clusters of activity in the right occipital pole, the left precentralgyrus, the right superior parietal gyrus, the left superior temporal gyrus, the left SMA and the left middle temporal gyrus. The opposite contrast (M1+ > HC) showed clusters of activity bilaterally in the occipital cortex, bilaterally in the lateral occipital cortex, in the right suparmarginalgyrus, the right precentralgyrus, the left inferior temporal gyrus and the right angular gyrus (Table 5, Figure 3A).

**Table 5:** **MNI coordinates and group statistic contrasts for voxels that were most strongly activated by verbs generation task vs. rest**.

			**Coordinates (mm)**								**Coordinates (mm)**				
**Cluster**	**Voxels**	**P**	***Z***	***x***	***y***	***z***		**Cluster**	**Voxels**	***P***	***Z***	***x***	***y***	***z***	
**HC > M1−**								**M1− > HC**							
13	4479	1.75E-43	17.2	−10	−102	−2	Occipital pole L	7	1559	1.39E-20	9.72	−44	−72	−2	Lateral occipital cortex L
12	1977	1.97E-24	5.87	−40	0	62	Middle frontal gyrus L	6	918	6.56E-14	8.64	26	−88	20	Lateral occipital cortex R
11	996	8.64E-15	5.38	54	34	10	Inferior frontal gyrus, pars triangularis R	5	358	1.43E-06	8.02	22	−82	−2	Occipital fusiform gyrus R
10	869	2.41E-13	5.91	−6	14	70	Superior frontal gyrus(SMA) L	4	186	0.00138	7.03	−58	0	38	Precentral gyrus L
9	755	5.53E-12	5.62	30	−2	62	Superior frontal gyrus R	3	161	0.00434	4.3	−52	34	2	Inferior frontal gyrus, pars triangularis L
8	574	1.13E-09	6.64	−52	18	38	Middle frontal gyrus L	2	159	0.00477	5.37	−54	−38	16	Planumtemporale L
7	470	5.96E-08	4.45	70	−34	−12	Middle temporal gyrus R	1	117	0.0377	5.66	10	−94	24	Occipital pole R
6	465	5.96E-08	5.92	42	46	28	Precentral gyrus R								
5	238	0.000147	4.07	16	60	26	Frontal pole R								
4	211	0.00046	4.46	−68	−22	−6	Middle temporal gyrus L								
3	207	0.000546	5.03	−36	46	−8	Frontal pole L								
2	155	0.00576	5.1	−58	14	2	Inferior frontal gyrus L								
1	130	0.0195	6.16	−40	−76	46	Lateral occipital cortex, angular gyrus L								
**HC > M1+**								**M1+ > HC**							
18	5394	0	14.8	16	−102	18	Occipital pole R	9	3090	5.39E-33	11.4	26	−94	28	Occipital pole R
17	875	3.61E-13	6.66	60	0	48	Precentral gyrus R	8	711	3.16E-11	7.57	20	−82	54	Lateral occipital cortex R
16	797	2.91E-12	9.98	−46	6	34	Middle frontal gyrus L	7	475	5.96E-08	6.47	−24	−74	54	Lateral occipital cortex L
15	591	1.04E-09	6.87	2	−2	72	Justapositional lobule cortex (SMA) R	6	355	2.26E-06	4.59	−66	−20	14	Postcentral gyrus L
14	544	4.35E-09	7.36	−40	−76	46	Lateral occipital cortex L	5	176	0.00268	6.03	58	−56	0	Middle temporal gyrus R
13	411	2.98E-07	6.8	−22	−56	54	Superior parietal lobule L	4	175	0.0028	6.81	44	4	40	Precentral gyrus R
12	361	1.79E-06	7.25	22	−76	−14	Occipital fusiform gyrus R	3	150	0.00887	5	−12	−54	76	Superior parietal lobule L
11	319	8.29E-06	4.78	−60	12	4	Inferior frontal gyrus, pars opercularis L	2	128	0.0257	4.03	58	−38	32	Supramarginal gyrus R
10	260	7.85E-05	6.34	48	2	30	Precentral gyrus R	1	115	0.0492	8.2	−60	−14	38	Postcentral gyrus L
9	237	0.000198	4.72	−26	−4	54	Middle frontal gyrus L								
8	221	0.000382	4.36	22	32	50	Superior frontal gyrus R								
7	211	0.000582	6.07	58	36	6	Frontal pole R								
6	169	0.00367	4.62	0	64	18	Frontal pole R								
5	151	0.00846	5.09	56	22	−18	Temporal lobe R								
4	150	0.00887	4.91	6	−4	30	Cingulate gyrus R								
3	140	0.0143	4.31	−52	−48	−6	Middle temporal gyrus L								
2	133	0.0201	5.07	−64	−54	20	Angular gyrus L								
1	122	0.0346	4.67	4	62	0	Frontal pole R								
**M1+ > M1−**								**M1− > M +**							
1	4762	0	9.39	28	−88	20	Lateral_occipital cortex R	1	655	3.03E-12	6.92	−40	−58	56	Parietal inferior gyrus L
2	408	2.06E-08	5.39	52	−4	50	Precentral gyrus R	2	238	2.71E-05	6.29	60	−64	0	Lateral occipital cortex R
3	323	6.56E-07	5.26	48	2	28	Precentral gyrus R	3	233	3.42E-05	6.91	26	−62	36	Occipital superior cortex R
4	303	1.49E-06	4.34	−52	28	−4	inferiorfrontal gyrus L	4	209	0.000107	5.79	28	−54	70	Superior parietal gyrus R
5	214	8.42E-05	4.6	−44	8	−12	Temporal pole L	5	187	0.000318	4.17	−62	−24	24	Supramarginal gyrus L
6	204	0.000137	4.96	−28	−52	54	Superior parietal lobule L	6	158	0.00143	7.68	22	−66	68	Superior parietal gyrus L
7	200	0.000167	8.31	−58	2	38	Precentral gyrus L	7	134	0.00531	5.07	−22	−78	50	Superior parietal gyrus R
8	152	0.00197	5.38	44	−86	6	Lateral occipital cortex L	8	130	0.00666	4.87	−28	−72	30	Midde occipital gyrus L
9	122	0.0105	5.05	26	−54	46	Angular gyrus L	9	125	0.00885	6.01	54	−70	20	Middle temporal gyrus L
10	106	0.027	4.25	−42	14	50	Middle frontal gyrus L	10	124	0.00938	4.18	6	−10	64	SMA A
								11	116	0.0149	6.56	48	−84	14	Midde occipital gyrus L
								12	108	0.024	4.56	−28	40	36	Middle frontal gyrus L
								13	101	0.0366	5.18	42	44	28	Middle frontal gyrus L
								14	100	0.0389	11	−14	−102	4	Midde occipital gyrus L
								15	98	0.0439	6.76	40	−70	−4	Inferior temporal cortex R

HC, healthy controls; M1−, lesions involving the primary motor cortex; M1+, lesions sparing the primary motor cortex.

We tested the difference between BOLD signals extracted from the Broca's area seed and the motor area seed during the verb generation task from all three groups by running the ANOVA test. There were no significant differences between all three groups when we tested signals from the motor area [*F*_(2)_ = 0.586, *p* = 0.557]. Conversely, when the seed was centered on the Broca's area, we found a significant difference between (verb generation > rest) M1+ versus (verb generation > rest) HC and (verb generation > rest) M1− BOLD signals [*F*_(2)_ = 18.917, *p* < 0.001] (Figure 3B).

#### Functional connectivity analysis with psycho-physiological interactions (PPI)

For all three groups [healthy controls (HC), the group with lesions involving M1 (M1−), the group with lesions sparing M1 (M1+)], there were no regions exhibiting significant functional connectivity depending on the seed activity when it was extracted from the primary motor cortex (see Figure 2C). When the seed ROI was centered on the inferior frontal gyrus (Broca's area), the PPI analysis on healthy participants showed that the verb generation task increased the functional connectivity with a cluster overlapping bilaterally the angular gyrus (*Z* = 4.2 right, *Z* = 3.86 left), the left middle frontal gyrus (*Z* = 3.98), the left frontal pole (*Z* = 3.72), the left posterior cingulate gyrus (*Z* = 4.08), the left putamen (*Z* = 4.59) and the middle temporal gyrus (*Z* = 3.13) (Table 6, Figure 2B). For the M1− group, the analysis showed an increased connectivity between the inferior frontal gyrus (Broca's area) and the right posterior brain areas, specifically the inferior occipital cortex (*Z* = 5.03), the calcarine cortex (*Z* = 3.71), the temporal inferior cortex (*Z* = 3.62) and the fusiform areas (*Z* = 3.46).

**Table 6:** **Brain regions showing significant increases of connectivity to the left Boca's area during the verb generation task vs. rest as revealed by the PPI analysis**.

				**Coordinates (mm)**			
**Cluster**	**Voxels**	***P***	***Z*-value**	***x***	***y***	***z***	
**HC**							
9	436	6.30E-09	4.09	−54	−66	22	Lateral occipital Cortex L
			3.28	−56	−62	40	Angular gyrus L
8	408	1.87E-08	4.25	58	−60	32	Lateral occipital cortex R
			4.06	60	−60	28	Angular gyrus R
			3.31	48	−68	36	Lateral occipital cortex R
7	205	0.00013	3.86	−48	16	48	Middle frontal gyrus L
			3.45	−50	22	40	Inferior frontal gyrus, pars opercularis L
6	201	0.000159	4.07	−6	52	48	Frontal pole L
			3.32	−10	56	36	Superior frontal gyrus L
5	198	0.000185	3.91	−46	32	−24	Frontal orbital cortex L
			3.81	−48	28	−12	Frontal operculum cortex L
			3.54	−48	38	−16	Inferior frontal gyrus, pars triangularis L
4	162	0.00119	4.11	−42	52	0	Frontal pole L
			4.11	−42	52	0	Frontal pole L
3	137	0.00467	3.6	−72	−38	−12	Middle temporal gyrus L
			3.14	−66	−36	−6	Superior temporal gyrus L
			2.92	−60	−38	−6	Middle temporal gyrus, temporoccipital *p*. L
2	136	0.00494	3.94	−24	24	6	Insular cortex L
			3.58	−20	32	−10	Fronto orbital cortex L
1	121	0.0117	3.82	−4	−40	34	Cingulate gyrus L
			2.65	2	−36	36	Cingulate gyrus, precuneous R
			2.53	−4	−42	44	Precuneous cortex L
**M1−**							
1	515	6.26E-06	5.03	42	−74	−4	Inferior occipital cortex R
			3.71	12	−92	4	Calcarine cortex R
			3.62	46	−64	−6	Temporal inferior cortex R
			3.46	28	−78	−2	Fusiform gyrus R

HC, healthy controls; M1−, lesions involving the primary motor cortex.

PPI analysis on the M1+ group did not display any brain area showing significant task-specific correlation to the seed ROI on the inferior frontal gyrus (Broca's area) at the predefined threshold.

## Discussion

This study was designed to further explore the nature of the interaction between the M1 cortex and linguistic processing. Specifically, we investigated patients' proficiency in performing a verb naming task and we analyzed the functional connectivity between language-related areas and the M1 cortex during a verb generation task. The verb naming task has been widely used in neuroimaging studies of language to explore the lexico-semantic features of the language network (Demonet et al., 2005; Peran et al., 2009). Previous studies addressed the neural correlates of verb generation in healthy participants (Crescentini et al., 2010; Peran et al., 2010) and in Parkinson disease patients (Peran et al., 2009). However, none addressed the functional connectivity in neurosurgical patients who show a decreased ability in processing action-related words, in patients who are proficient and in healthy controls.

Our main finding is a proficient verb naming performance of patients whose lesion involved M1, a degraded verb naming performance of patients whose lesion spared M1, and a lack of significant changes in the functional coupling between the left M1 cortex and other brain areas during the verb generation task both for healthy controls and for patients. Before we address the implications of our main finding, we first discuss results concerning the anatomo-functional correlates of the action-verb generation task in healthy participants and in neurosurgical patients with lesions involving the motor system and the main differences between their activations under classical General Linear Model assumptions. The task-related network reflected language processing; the activations encompassed areas which have been shown by fMRI and PET studies to be involved in semantic processing (e.g., Tettamanti et al., 2005; Peran et al., 2010; Esopenko et al., 2012); areas reflecting language processing were the ventral occipital cortex bilaterally extending to the left anterior superior temporal gyrus and the left TPJ; areas activated in conjunction with bilateral activations of the premotor cortex were found bilaterally in the superior parietal cortex and in the left intraparietal sulcus. These findings confirm earlier reports of a general role of these areas in semantic processing (Chao and Martin, 2000; Price, 2000). The activation of the left inferior frontal region (despite the presence of glioma) and not in right homologue regions rule out the possibility of long-term shifts of function which are typically found in low grade glioma but not in high grade glioma (Thiel et al., 2001; Duffau et al., 2003; Thiel et al., 2005; Keidel et al., 2010). In addition, that semantic processing related areas were activated by the verb generation task is consistent with previous studies that have emphasized graded differences between verbs and nouns in terms of imageability, contextual diversity, etc. (e.g., Bird et al., 2000). Additional activation clusters, included the dorso-lateral prefrontal cortex bilaterally, most likely reflecting the supervisory demands of the task. To sum up, the task induced activation in fronto-temporal and temporo-occipital regions and the SMA as previously found (Peran et al., 2009). It is remarkable that, we did not find any activation in the primary motor area during the verb generation task, either in healthy controls and in patients. This was particularly evidenced by the analysis performed on the parameter estimates extracted from the ROI reconstructed on the M1 hand areas of each participant. In that analysis we found that both patients with a decreased performance in action verb naming and those who were proficient did not show any significant difference from the parameter estimates of healthy controls. By contrast, we found between groups differences in the analysis performed on the parameter estimates extracted from the ROI in the inferior frontal gyrus (Pars Opercularis) of each participant.

The analysis of the fMRI signal showed a consistent reduction on the intensity depending on the group of subjects: patients who had a spared action naming ability showed a significantly lower signal compared to healthy controls; patients with a decreased performance in action verb naming showed the lowest intensity of any group. This result indicates that, activation in M1 cortex is not a necessary component of the network of areas supporting the action verb generation task. Interestingly, we found that, with respect to healthy controls, patients with a lesion involving M1 and a spared verb naming (M1−) differentially activated the left middle temporal gyrus/angular gyrus, the left inferior frontal gyrus/pars triangularis and the left precentral cortex. By contrast, we found that with respect to patients with a lesion sparing M1 cortex and an impaired verb naming (M1+), healthy controls differentially activated the left superior temporal gyrus and bilaterally the middle temporal gyrus. In turn, M1− as compared to M1+ differentially activated the left supramarginalgyrus, bilaterally the middle temporal gyrus and the right inferior temporal gyrus, the left inferior parietal lobe and bilaterally the inferior parietal lobe, whereas M1+ as compared to M1− differentially activated bilaterally the precentralgyrus, the left superior temporal pole, the left inferior parietal lobe and the right angular gyrus. All these areas are key hubs associated to semantic processing, with the left precentralgyrus, especially related to the semantic processing of action related items (e.g., Tettamanti et al., 2005). In the case of M1−, their activation is interpreted here as likely being due to an increased effort required to perform the fMRI tasks, whereas in the case of M1+ a lack of activation in this area seems to indicate a correlation with the low performance in action naming.

In addition, with respect to M1− and to M1+, healthy controls differentially activated the superior and middle frontal gyrus. In turn, with respect to M1+, M1− differentially activated the middle frontal gyrus bilaterally, while M1+ activated the left middle and orbital frontal gyrus, as compared to M1−. This data indicate that healthy controls had more executive control-related resources available with respect to patients, as had M1− with respect to M1+ e.g., for functional interaction between associative retrieval and executive control, see (Crescentini et al., 2010). Healthy controls, with respect to M1+, differentially recruited the right precentralgyrus extending to the inferior frontal gyrus/pars opercularis, as previously found with the verb generation task (Papathanassiou et al., 2000). In addition, with respect to M1−, healthy controls differentially recruited the left postcentralgyrus since the patients' lesions often extended to the left postcentral area and consequently they lacked the BOLD signal from this area. Similarly, healthy controls with respect to M1+, differentially activated the SMA, as did M1− for the right SMA as compared to M1+ patients, since M1+ patients' lesions often extended to this area and consequently they lacked the BOLD signal from it. Lastly, with respect to M1−, controls differentially activated bilaterally the middle temporal gyrus, as previously observed in other studies (Esopenko et al., 2012). A last between-group difference involved the occipital lobe. With respect to M1−, healthy controls differentially activated bilaterally the lateral occipital cortex, which we realized was due to the different field of view we used during acquisition for the patient's groups, accidentally cutting the lower part of the occipital lobes. Indeed also M1−, and M1+, with respect to healthy controls, differentially activated the bilaterally lateral occipital cortex. The DTI analysis revealed that the superior longitudinal fasciculus (SLF) was reconstructed in all the patients. In particular, for the M1+, the DTI analysis revealed that the SLF was intact in one patient, infiltrated in another case, and displaced (but not damaged) in three cases. This suggests that parts of the lesions have probably involved white matter (as it is typical for glioma). However, for the M1+, the SLF was never found interrupted, therefore the possibility that disconnection syndromes as well as local cortical dysfunction could make the picture more complicated can be ruled out.

### Neuropsychological data

The strong version of the embodied theory of language processing proposes that the sensorimotor cortex is involved in the processing and representation of action-related items. Some theories suggest that sensorimotor areas are an integral part of lexical-semantic representations (Pulvermuller, 2005; Pulvermuller et al., 2005a,b). Others suggest that motor activations are flexible and context-dependent (Tomasino et al., 2010; van Dam et al., 2010; Tomasino et al., 2012; van Dam et al., 2012b). In our study, patients with lesions involving the M1 cortex had a performance within the normal range in action naming, whereas patients with lesions sparing the M1 cortex were impaired, confirming the view that lesions to M1 do not predictably cause deficits in action word processing (De Renzi and di Pellegrino, 1995; Saygin et al., 2004; Mahon et al., 2007; Negri et al., 2007; Mahon and Caramazza, 2008; Tomasino et al., 2012). Those who presented an impaired performance were patients with lesions sparing the M1 cortex. These results complement those reported in studies addressing the neural basis of semantic memory (e.g., Vandenberghe et al., 1996; Rogers et al., 2004; Pobric et al., 2007; Binney et al., 2010; Lambon Ralph et al., 2010; Visser and Lambon Ralph, 2011). A parallel distributed processing implementation of the *view* that suggests that semantic knowledge arises from the interaction of perceptual representations of objects and words has been tested in a computational model of semantic representation (Rogers et al., 2004), indicating that the representational hub is especially important for conceptual formation (Rogers et al., 2004). It has been argued that concept representations reflect the conjoint action of modality-specific sources of information, as well as a transmodal hub which is required in order to form “coherent” concepts (Lambon Ralph et al., 2010). However, it has also been put forward that neuropsychological data from patients with semantic dementia showed that damage to ventrolateral anterior temporal regions (and not to modality-specific association regions) generates a selective degradation of conceptual knowledge (Lambon Ralph et al., 2010). These clinical data from patients with semantic dementia in the context of focal atrophy of the anterior temporal lobe (Lambon Ralph et al., 2010) has been confirmed by a repetitive transcranial magnetic stimulation (rTMS) of the anterior temporal lobe (Pobric et al., 2007). Authors showed that rTMS over the left anterior temporal lobe significantly increased naming latencies for a specific-level naming task but not for number naming, and significantly slowed synonym judgment times but not number quantity decisions (Pobric et al., 2007). Lastly, fMRI data (e.g., Vandenberghe et al., 1996; Binney et al., 2010; Visser and Lambon Ralph, 2011) and probabilistic tractography (e.g., Binney et al., 2012) further supported the role of the temporal cortex as a zone of gradual convergence of sensory information that culminates in modality and perceptually invariant representations found in the most rostral part of this area (Binney et al., 2012). Authors (Binney et al., 2012) explore the connectivity of specific temporal lobe areas to frontal and parietal language regions, and among the regions they found to be connected to the temporal areas, no evidence of connections to M1 cortex or premotor area was found. Similarly, our neuropsychological and fMRI_PPI results suggest that sensorimotor areas are not invariantly involved in the semantic processing and representation of action-related items. To rule out the possibility that the lack of connectivity between the Broca's area and the M1 in M1+ was due to the difficulties that those participants had in generating verbs to pictures of objects, we considered that the lack of connectivity between the Broca's area and the motor system was found also for M1−, whose performance at verb naming is within the normal range, ensuring thus that they properly carried out the task in the scanner. The same result was found in healthy controls. In addition, as a confirmation that the M1+ could produce the verbs used in the experiment, we verified that 50% of the items included in the list of items used in the verb generation fMRI task were part of the neuropsychological verb naming task [B. A. D. A.: A Battery for the assessment of aphasic disorders] (Miceli et al., 1994). Taken together our results contribute to the embodied cognition debate. Supporters of the strong version of this view hypothesize that the M1 area is necessary for the semantic analysis of an action-related word item; however, in our study, a damaged M1 area did not cause a degraded verb naming performance. In particular, the disembodied view argues that the motor system may be activated during action-word processing but not necessarily so (Mahon and Caramazza, 2005, 2008). This view is in line with the notion of flexibility in language representation whereby the degree to which a modality specific region contributes to a representation depends on the context (van Dam et al., 2010, 2012b) in which conceptual features are retrieved. Flexibility is characterized by the relative presence or absence of activation in motor and perceptual brain areas. Our results are also in accordance with the idea of a top down modulation exerting its influence in selecting the type of strategy adopted while processing language, according to which different strategies can cause participants to lean on different sorts of sensorimotor representations (Tomasino and Rumiati, 2013). Therefore, our data provides support to the idea that the activation of the M1 area may not be absolutely necessary for language comprehension.

### Psycho-physiological interactions (PPI)

We measured the functional connectivity between language-related areas and motor-related areas as calculated by psycho-physiological interactions (PPI) in healthy controls and in patients whose lesion affected M1. PPI analysis aimed to explain neural responses in one brain area in terms of the interaction between influences of another brain region and a cognitive process (here: action-related word processing). As a first result we found that the M1 cortex did not show an essential role, since, when the seed for the PPI analysis was positioned in it, this area was not significantly changing its functional connectivity to any nodes of the linguistic network. In addition, by positioning the seed for the PPI analysis in the inferior frontal gyrus, the motor area was not part of the network of areas which were significantly changing their functional connectivity to the inferior frontalgyrus. In more detail, PPI analyses performed with the left primary motor area (as revealed by the motor localizer task) as seed assessed the areas with increased connectivity with the left primary motor area and with the left inferior frontal gyrus during action-related word processing. No significant changes in the functional coupling between the left primary motor area and other brain areas were observed both in healthy controls and in patients during action-related word processing. This result is in contrast with previous studies addressing how do language-related areas and motor-related areas functionally talk to each other (Tettamanti et al., 2008; Ghio and Tettamanti, 2010). Those studies showed that the degree of functional integration between the left inferior frontal gyrus and the left fronto-parieto-temporal system, including the dorsal premotor cortex, the supramarginalgyrus, and the left posterior inferior temporal gyrus, was more positive for processing action-related vs. abstract sentences (Tettamanti et al., 2008). Moreover, the same studies showed that when participants processed action-related sentences during the listening of either action-related or abstract sentences, the left superior temporal gyrus was more strongly connected with the left-hemispheric action representation system, including sensorimotor areas, while the left inferior-ventral frontal, temporal, and retrosplenial cingulate areas were activated when processing abstract sentences (Ghio and Tettamanti, 2010).

Our results, on the other hand, are consistent with a previous psycho-physiological interaction (PPI) study, in which authors corroborate the view that language representations are flexible and context-dependent (van Dam et al., 2012a). In our study, generating action verbs associated with a target object did not automatically activate M1 and did not increase functional connectivity to this area in healthy controls and both in patients that were proficient and those who showed an impaired performance at verb naming.

PPI analyses performed with the left inferior frontal gyrus (Pars Opercularis, revealed by the whole brain analysis of the main fMRI experiment) as seed assessed the areas with increased connectivity with the left inferior frontal gyrus during action-related verb processing. Results showed that in healthy controls the verb generation task increased connectivity between the left inferior frontal gyrus and an extensive network, including the inferior left middle frontal gyrus, the left middle orbital gyrus/frontal pole associated with executive control triggered during verb generation and the left inferior frontal gyrus (pars triangularis), the left putamen, the angular gyrus bilaterally and the left middle temporal gyrus, associated with semantic retrieval and semantic knowledge (Mahon and Caramazza, 2008). It is known that the regions involved in naming tools and other artifacts include the left posterior middle temporal gyrus (pMTG), the bilateral inferior temporal gyri, the left middle temporal gyrus, and the left premotor region (Martin et al., 1996). The relative contribution of the various areas may vary depending on the type of task used (Martin et al., 1996; Tyler and Moss, 2001) [For reviews see (Martin et al., 1996; Mahon and Caramazza, 2009)]. In most current models of language representation, temporal lobe regions have been implicated in aspects of semantic processing, for reviews see (e.g., Martin and Chao, 2001; Binder et al., 2009). Some authors, for example, posit the angular gyrus and anterior inferior temporal regions as key in semantic processing, while others (Indefrey and Levelt, 2004) suggest that lemma retrieval and selection occur in the middle temporal gyrus, or others (Hickok and Poeppel, 2004, 2007) propose a role for the bilateral posterior middle and inferior portions of the temporal lobe corresponding to the lexical interface, which is seen to link phonological and lexical (including semantic) information. In this context, our PPI analysis showed that in healthy controls our task increased the functional connectivity between the inferior frontal gyrus and two essential nodes of the semantic system.

As a second result, we found that in the M1− patients, the verb generation task increased connectivity between the left inferior frontal gyrus and the left lateral occipito-temporal cortex. Previous studies investigating category selective responses by using DCM (Noppeney et al., 2006), showed that the occipito-temporal gyrus was one of the nodes with increased functional connectivity during tool processing. Interestingly, in our study we found that one cluster was localized close to the coordinates of the occipito-temporal/extra-striate cortex, i.e., a complex brain region that processes not only body parts, but also motion and tools (Gitelman et al., 1999; Huk et al., 2002; Downing et al., 2007; Bracci et al., 2010; Kolster et al., 2010; Valyear and Culham, 2010; Bracci et al., 2012); for a review see (Weiner and Grill-Spector, 2011). It has been shown that these body-related occipito-temporal/extra-striate areas are modulated by the simulated use of appropriate tools (Tomasino et al., 2012). Lastly, we found that no significant changes in the functional coupling between the left inferior frontal gyrus and other brain areas were observed during the verb generation task in M1+ patients. This is consistent with the presence of an action naming decrease at behavioral level. Both groups of patients had a different pattern of connectivity with respect to controls. In a study on neurosurgical patients it has been suggested that the effect of brain lesions may be better evaluated over the entire network rather than on the basis of the activity of isolated regions (Briganti et al., 2012). Authors positioned the seed in the inferior frontal gyrus and tested for differences in functional connectivity between patients and controls during a verb generation task and showed that patients had a reduced functional connectivity of the language network. Remarkably, it has been shown that the reduction was not confined to the area surrounding the tumor, but also involved remote areas of the contralateral hemisphere. In particular, similarly to our results, there is evidence that patients showed a decreased bilateral connectivity in the temporo-parietal area (TPJ) (Briganti et al., 2012). Authors underlined the crucial role of TPJ area for the integrity of functional networks and suggested a particular vulnerability of this area to local and non-local disturbances (Briganti et al., 2012).

Taken together, our results suggest that the activation of the M1 cortex is not as automatic as held by the strong version of the embodied accounts of language processing, suggesting that sensorimotor areas are involved in the processing and the representation of action-related items. In our study, patients with lesions involving the M1 cortex had a performance within the normal range in action naming. In addition, both for healthy controls and for patients (either those with a spared and those with an impaired verb naming performance) no significant changes in the functional coupling between the left motor cortex and other brain areas were observed during the verb generation task. Moreover, patients with a lesion in M1 showed that the verb generation task increased connectivity between the left inferior frontal gyrus and the left EBA, an area which has been related to motor imagery of tool use, and the occipito-temporal cortex related to semantic processing of tools.

To conclude, PPI detects regions whose activation could be explained by the activation pattern of a seed region in interplay with a specific cognitive or sensory process. For this reason, the lack of functional connectivity changes between the left inferior frontal gyrus and M1 would support the view that sensory-motor activity is not necessary but rather accessory to linguistic processing (Tomasino et al., 2007; Mahon and Caramazza, 2008; Postle et al., 2008; Tomasino et al., 2008; Papeo et al., 2009; Raposo et al., 2009; Tomasino et al., 2010; Willems et al., 2010; Papeo et al., 2012b; Tomasino et al., 2012).

### Conflict of interest statement

The authors declare that the research was conducted in the absence of any commercial or financial relationships that could be construed as a potential conflict of interest.

## Appendix

List of items included in the verb generation fMRI task and in the neuropsychological battery (verb naming). In bold type the verbs of the fMRI experiment matched with verbs used in the neuropsycological experiment.

**Table:**

**Verb generation fMRI task**	**BADA stimuli**
**Bicchiere (glass)**	**Versare (to pour)**
**Chitarra (guitar)**	**Suonare (to sing)**
**Fucile (rifle)**	**Sparare (to shoot)**
**Fiasco (flask)**	**Versare (to pour) rep**
**Violino (violin)**	**Suonare (to sing) rep**
**Telefono (telephon)**	**Telefonare (to call)**
**Fisarmonica (accordion)**	**Suonare (to sing) rep**
**Forbici (scissors)**	**Tagliare (to cut)**
**Gelato (ice cream)**	**Leccare (to lick)**
**Martello (hammer)**	**Scolpire (to sculpt)**
**Pala (shovel)**	**Scavare (to dig)**
**Sega (saw)**	**Segare (to saw)**
Automobile (car)	Fischiare (to whistle)
Annaffiatoio (watering can)	Annegare (to drown)
Scala (stairway)	Bussare (to knock)
Chiave (key)	Correre (to run)
Ago (needle)	Accendere (to light)
Pattini (skate)	Scavalcare (to climb over)
Penna (pen)	Cancellare (to delete)
Pettine (comb)	Baciare (to kiss)
Righello (ruler)	Indicare (to indicate)
Scopa (broom)	Giurare (to take an oath)
Corda ( rope)	Seminare (to sow)
Bicicletta (bicycle)	Stirare (to iron)
	Spingere (to push)
	Applaudire (to clap)
	Costruire (to build)
	Dormire (to sleep)
	Azzannare (to snap at)
	Sudare (to sweat)
	Nuotare (to swim)
